# Pedicle distraction increases intervertebral and spinal canal area in a cadaver and bone model

**DOI:** 10.1051/sicotj/2018009

**Published:** 2018-05-04

**Authors:** Matthew Hughes, Nikolaos Papadakos, Tim Bishop, Jason Bernard

**Affiliations:** 1 St Georges, University of London, Cranmer Terrace, London SW17 0RE UK; 2 Department of Radiology, St Georges Hospital, Blackshaw Road, London SW17 0QT UK; 3 Department of Orthopaedics, St Georges Hospital, Blackshaw Road, London SW17 0QT UK

**Keywords:** Lumbar spinal stenosis, Pedicle distraction, Pedicle osteotomy

## Abstract

*Introduction*: Lumbar spinal stenosis is degenerative narrowing of the spinal canal and/or intervertebral foramen causing compression of the spinal cord and nerve roots. Traditional decompression techniques can often cause significant trauma and vertebral instability. This paper evaluates a method of increasing pedicle length to decompress the spinal and intervertebral foramen, which could be done minimally invasive.

*Methods*: Three Sawbone (Sawbones Europe, Sweden) and 1 cadaveric lumbar spine underwent bilateral pedicle distraction at L4. A pedicle channel was drilled between the superior articular process and transverse process into the vertebral body. The pedicles underwent osteotomy at the midpoint. Screws were inserted bilaterally and fixated distraction of 0 mm, 2 mm, 4 mm and 6 mm. CT images were taken at each level of distraction. Foramen area was measured in the sagittal plane at L3/4. Spinal canal area was measured at L4 in the axial images. The cadaver was used to evaluate safety of osteotomy and soft tissue interactions preventing distraction. Statistical analysis was by student paired t-test and Pearson rank test.

*Results*: Increasing distraction led to greater Spinal canal area. From 4.27 cm^2^ to 5.72 cm^2^ (*p* = 0.002) with 6 mm distraction. A Maximal increase of 34.1%. Vertebral foramen area also increased with increasing pedicle distraction. From 2.43 cm^2^ to 3.22 cm^2^ (*p* = 0.022) with 6 mm distraction. A maximal increase of 32.3%. The cadaver spinal canal increased in area by 21.7%. The vertebral foramen increased in area by 36.2% (left) and 22.6% (right).

*Discussion*: For each increase in pedicle distraction the area of the spinal and vertebral foramen increases. Pedicle distraction could potentially be used to alleviate spinal stenosis and root impingement. A potential osteotomy plane could be at the midpoint of the pedicle with minimal risk to nerve roots and soft tissue restrictions to prevent distraction.

## Introduction

Lumbar spinal stenosis is a condition characterised by the narrowing of the spinal canal, vertebral foramen and lateral recess causing neural compression. Patients may present with a variety of symptoms such as: neurogenic claudication, radicular pain, generalized lower back pain, weakness and sensory disturbance [1]. Over 200 000 people in the United States are affected with lumbar spinal stenosis [2] of which the most common cause is degenerative change. Degenerative lumbar spine is the leading cause of spinal surgery amongst the over 65's [2], and is a major cause of morbidity amongst that group [1]. With more and more patients undergoing surgical intervention every year [3] and changing population demographics degenerative spinal disease is set to become a larger burden.

Lumbar spinal stenosis is caused by a combination of anatomical factors; disc prolapse and bulging, facet joint and ligamentum flavum thickening, osteophyte formation and endplate degeneration amongst them [1–4]. These can occur in isolation or most often as a combination [5].

The intervertebral foramen dimensions are influenced by the surrounding structures of the spinal complex. The intervertebral disc height has the greatest impact on the vertical dimensions [6]. With progressive disc height loss, as seen in ageing and degenerative spinal disorders, there is an increasing reduction on intervertebral area [7]. In the sagittal plane, it is the length of the spinal canal and pedicle which influences dimensions [6]. Thus, increasing the length of the pedicle surgically will potentially increase the area of the foramen and have a decompressive effect.

In spinal canal stenosis, the primary factors are not well understood. Some studies have concentrated on the morphology of the canal itself while others the degenerative changes, that of the disc and ligamentum flavum [1,5]. A combination of these factors likely leads to a reduced AP diameter of the spinal canal [5]. The pedicle is one the influencing structures in AP length of the spinal canal and thus increasing it would potentially decompress the Dural sac in the sagittal plane.

The lateral recess is bordered by the pedicle laterally, the articular facets posteriorly and the vertebral endplate and disc anteriorly [4]. As with other types of spinal stenosis changing the pedicle length will influence the dimensions of the affected anatomical area.

Current operative treatment is based upon the goal of decompression of the entrapped neural elements. Various procedures have been described such as conventional laminectomy, uni/bilateral laminotomy and laminoplasty [8]. However, there remains complications with open approaches, with increased rates of spondylolisthesis reported, amongst others [9].

Minimally invasive surgery in Lumbar stenosis has been shown to reduce hospital stays and operating time. Despite this there remains no conclusive evidence as to outcomes, pain or function scores compared to conventional treatments [10]. Micro decompression involves leaving intact the posterior structures with minimal bone and flavum removal. It has equivalent reported outcomes while reducing post-operative complications [8].

We look at the effects of increasing pedicle length on the vertebral and spinal foramen area as proof of concept for this potential minimally invasive technique. The study is the first to our knowledge to identify a safe osteotomy site and macroscopically assess soft tissue restrictions.

## Methods

### Sawbone study

Three Sawbone Lumbar Spines, (Sawbones Europe, Sweden) that include L3/4 vertebral level, were selected. Two specimens were L1- L5 and the other L2- S5.

A pedicle channel was created by drilling through the L4 pedicle into the vertebral body. The starting point was the intersection between the articular processes and transverse process.

The pedicles underwent osteotomy at the midpoint of the pedicle in the sagittal plane, perpendicular to the longitudinal axis.

**Figure 1:**
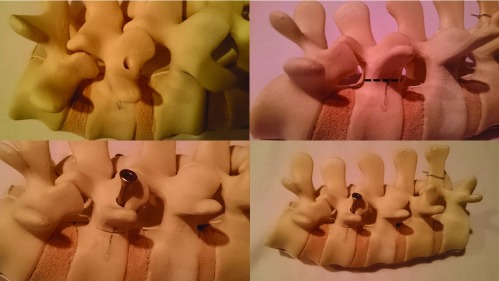
Sawbones spine (clockwise from top left): location of pedicle channel, location of osteotomy (dotted line), screw insertion following osteotomy, whole specimen.

The disarticulated spines were fixated with standard 5 × 70 mm wood screws, inserted bilaterally into the pedicle channels (Figure 1).

Each spine underwent sequential distraction of 0 mm, 2 mm, 4 mm and 6 mm at the site of pedicle osteotomy. Distraction was held with the insertion of spacers and fixated with the screws.

**Figure 2:**
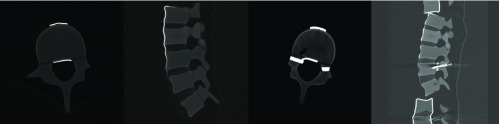
CT scanning of Sawbone spines: (from left to right) axial of control spine, sagittal control spine, axial image of distracted spine, sagittal image of distracted spine.

The Sawbone spines underwent CT scanning for each level of distraction (Figure 2).

Foramen area and diameter was measured at L3/L4 for the intervertebral foramen in the sagittal images and L4 for the spinal canal in the axial images.

### Cadaveric study

A single fresh frozen female cadaver (>75 years old) that had been cut at the L1/L2 disc and bilaterally through the neck of femur was prepared for the study as follows:

Dissection of the Abdomen and pelvis was carried out to isolate the lumbar spine and sacrum. The nerve roots were left undamaged and removed from the soft tissue tethers holding them in place. The fully dissected spine left the joint capsule, spinal ligaments and nerves all intact. Sacro-iliac joint was cut through to leave an isolated lumbo-sacral specimen.

A pedicle channel was created bilaterally at L4, with a free hand drilling technique [11]. The channel was located at the intersection of the transverse process and the line between articular processes, through the L4 pedicle into the vertebral body.

Osteotomy was at the midpoint of the pedicle, perpendicular to the longitudinal axis.

**Figure 3:**
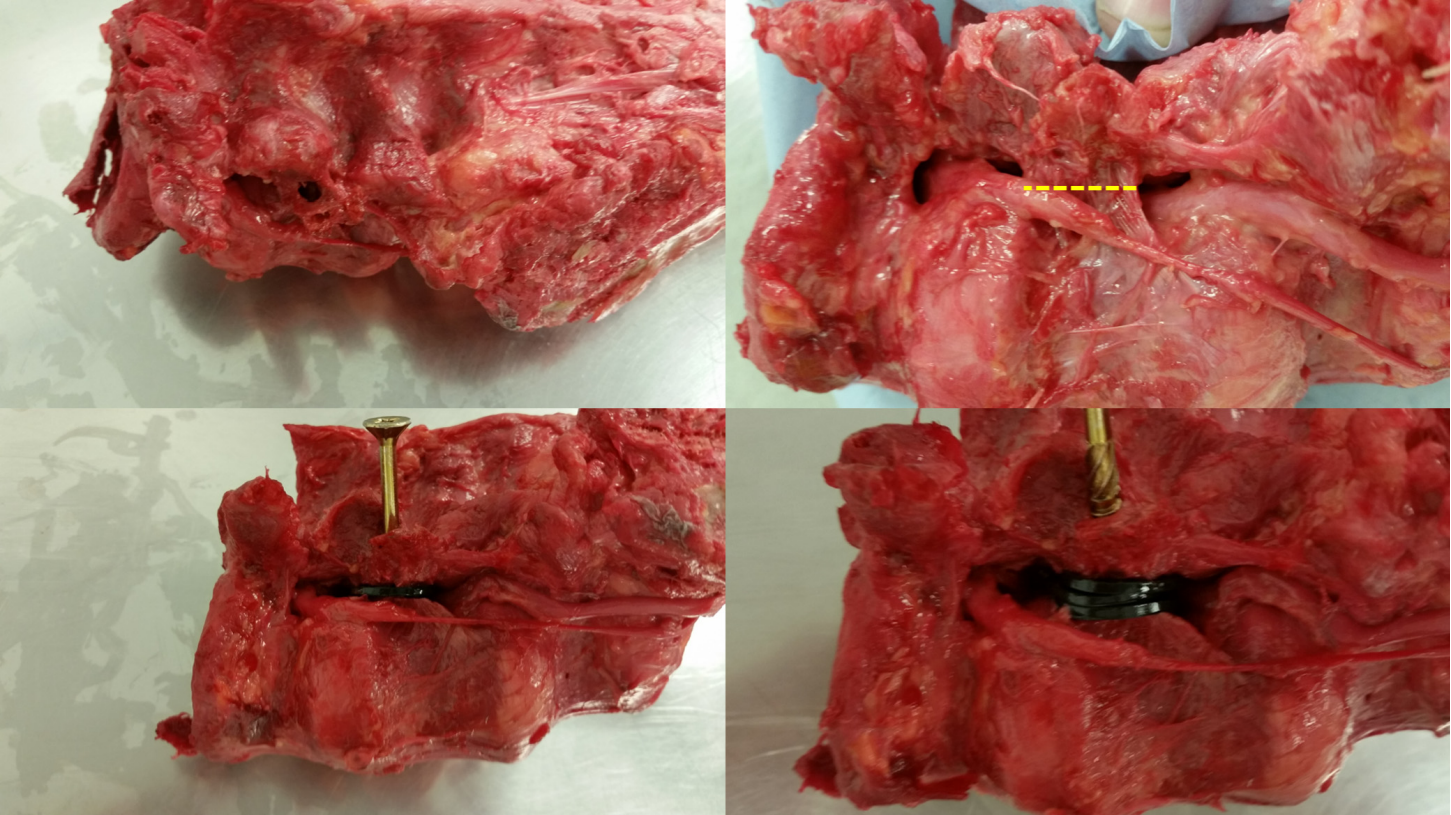
Cadaveric Spine (clockwise from top left): pedicle channel location, location of osteotomy along dotted line, pedicle screw insertion following 2 mm distraction, distraction of 6 mm.

The osteotomy was fixated with insertion of 5 × 70 mm wood screws. The spines were distracted at the osteotomy site. Degree of distraction was measured with a graphite calliper and the distraction maintained with spacers (Figure 3).

The spine underwent CT scanning in the sagittal and axial planes with 0, 2, 4 and 6 mm of distraction. Measurements of vertebral foraminal area were taken at the L3/L4 disc level and area of the spinal canal at the L4 vertebral body.

After CT scanning, the cadaver was analysed for Dural damage and tethering of the nerve roots during distraction. All pedicles underwent osteotomy and with gentle manual distraction the posterior column was removed from the specimen. Care was taken to allow the neural components to fall naturally and blunt dissection of soft tissue tethers released the dura from the posterior component. Integrity of the dura was found by injection of normal saline into the open dura superiorly and watching for lateral leakage around the osteotomy level.

### CT measurement

Measurements in the axial plane were taken as follows: Axial slice at the level of the pedicle osteotomy, L4 vertebral body. Free hand measurement was made on PACS to calculate the spinal canal area. Each specimen was measured 3 times, the image reset between each measurement and the average result was taken as the area of the spinal canal.

In the sagittal plane: Sagittal slice corresponding to the sagittal axis of the pedicle was used. Free hand measurement tool on PACS was used to draw around the foramen at l3/l4 vertebral level. Three measurements were taken with the image reset between each measurement. The average measurement was used for the area of the intervertebral foramen.

### Statistics

Statistical analysis was by Student paired t-test and Pearson rank test

## Results

### Sawbones

As pedicle length increased the cross-sectional area of both the spinal and intervertebral foramen increased.

**Figure 4:**
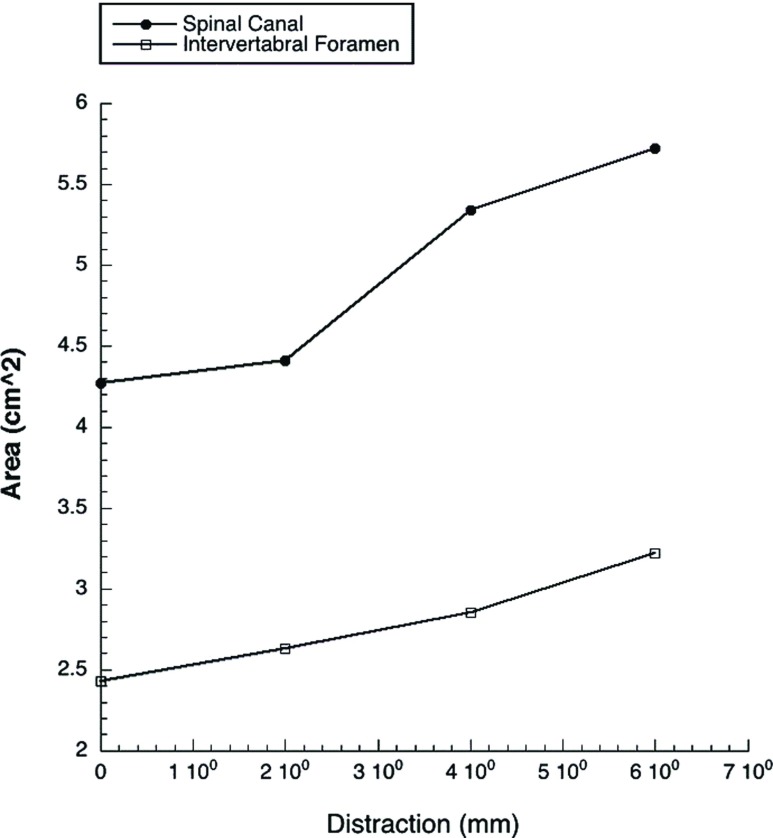
Distraction of pedicles plotted against area of spinal and intervertebral foramen. With a trend to towards increasing area with increased distraction.

The spinal canal cross-sectional area at 0 mm of distraction was 4.27 mm^2^, at 2 mm − 4.41 mm^2^, at 4 mm − 5.34 mm^2^, and at 6 mm − 5.72 mm^2^ (Figure 4). A maximal increase in area of 34% (*p* < 0.05).

At 0 mm of distraction the intervertebral foramen had a sagittal cross-sectional area of 2.43 mm^2^, at 2 mm − 2.63 mm^2^, at 4 mm − 2.82 mm^2^, and at 6 mm − 3.22 mm^2^ (Figure 4). A maximal increase in area of 32% (*p* < 0.05).

### Cadaver

With increasing distraction, the area of both the spinal canal and intervertebral foramen increased.

The spinal canal increased from 4.12 cm^2^ to: 4.35 cm^2^ at 2 mm of distraction, 4.68 cm^2^ at 4 mm and 5.01 cm^2^ at 6 mm. This represents a 5.70%, 13.80% and 21.70% increase in area respectively.

The intervertebral foramen area followed the same trend of increased area with distraction. However, there were differences between both the left and right intervertebral foramen with regards to absolute and relative increase of area.

The left foramen increased from 2.48 cm^2^ to: 2.97 cm^2^, 3.06 cm^2^ and 3.38 cm^2^ at 2, 4 and 6 mm of distraction. A relative increase of 19.70%, 23.30% and 36.20%.

The right foramen increased from 1.95 cm^2^ to: 2.11 cm^2^, 2.25 cm^2^ and 2.39 cm^2^ at 2, 4, and 6 mm of distraction as with previous measurements. A change in area of 8.30%, 15.70% and 22.60%.

### Dissection and anatomy

The cadaver had the benefit of having intact articular facets, capsules and cartilage. The dissection also left the posterior ligaments intact and unaltered. Minor dissection was only carried out around the neural fat in the intervertebral foramen for visualization of the nerve after CT measurements were taken. Gross appearances of the spine, showed some evidence of degenerative change. At the level of L4 the right facet joint showed hypertrophy and osteophyte growth. The left facet joint showed some degenerative change but was to a much lesser degree than the right. In our specimen, the intervertebral disc had some protrusion into the spinal canal and foraminal space.

The nerve root exited the foramen, reaching the narrowest point at the l3/l4 disc. It passed inferiorly along the posterolateral border of the disc and superiorly to the inferior pedicle at the junction between vertebral body and pedicle. The nerve continued in a posterior direction as it crossed the l4 pedicle. When making the pedicle osteotomy care must be taken at the junction of the vertebral body and pedicle in the supero-lateral aspect due to proximity to the descending nerve root. We found the best location of osteotomy to be at the midpoint of the pedicle. Osteotomy here allowed distraction as close to the vertebral body as possible, and thus the central mechanical axis, while avoiding the nerve root. Post distraction there appeared to be more space around the nerve root at its exit point and as it traversed the disc (Figure 3).

The major restraining forces came from the posterior articulations and ligaments. Increasing distraction required increasing force applied anteriorly towards the vertebral body and posteriorly to the distracted segment. However, without formal biomechanical testing the forces applied and the consequences of this are unknown.

**Figure 5:**
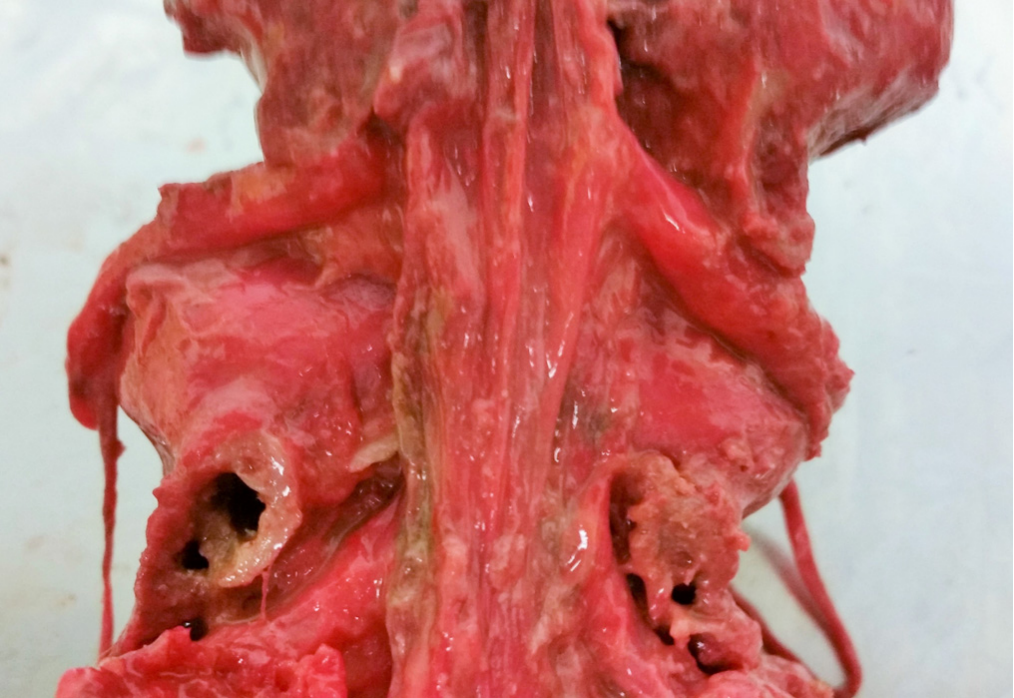
Dural integrity being examined.

After full distraction and removal of the posterior column the dura was inspected for damage during osteotomy and distraction (Figure 5). Saline injection revealed no Dural leakage around the levels of osteotomy and no tearing on the posterior surface due to tethering during distraction.

## Discussion

Our results indicate that by manipulation of the anatomical boundaries of the spinal and intervertebral canal the respective areas of the foramen will increase in a predictable manner. The increases in area between the spinal and intervertebral foramen were of similar magnitude, up to 34 and 32% respectively, indicating a potential decompression of both foramen with pedicle lengthening. Yuan et al. used a healthy 35-year-old male spine and increased the pedicle length at multiple spinal levels by up to 8 mm. Percentage increase of the spinal canal area was of comparable amounts across all lumbar levels, with a maximal increase seen at L2 of 66%. More variation was seen across the intervertebral foramen levels [12]. Much greater increase in size was noted in comparison to our study. Yet this is likely due to the degree of degenerative change encountered in our elderly spine compared to the healthy young male. Their results show the potential benefit at multiple levels of pedicle lengthening.

Spinal Stenosis often presents with multi-level disease. Kiapour et al. [13] looked at the effect of both single and bi-level lengthening of up to 4.5 mm. Noting substantive increases in spinal canal and neural foramen levels when combining L4 and L5 lengthening to decompress the L4-L5 spaces. Their results show how combining multiple levels of pedicle distraction could be possible for more complex disease.

Qian et al., looked at the use of pedicle lengthening for restoration of the spinal canal following . They noted that a mean pedicle lengthening of 2.17 mm was enough to restore the spinal canal volume to its original disc free diameter. They concluded that the required increase in pedicle length to restore canal volume could be predicted related to the amount of disc protrusion [14]. No work is yet to be done on quantifying the ideal amount of distraction at the pedicles in other causes of spinal stenosis. To translate the technique into clinical practise we must first identify a way to predict the required amount of distraction successfully.

During distraction of the cadaveric spine, it was noted that large forces were required to achieve full distraction at 6 mm. The force required to distract will cause significant AP force and shearing force across the disc space, and increased loading on the facet joints. No study to our knowledge has quantified these forces, and with this the risk of anterior slip of the vertebral body and spondylothesis.

Kiapour et al. tested pedicle lengthening of 4.5 mm in AP, lateral and axial movement planes. 10 nm moments in all directions produced no significant alteration in mechanics as compared to the intact spines. However, it was noted that an increase in intersegmental rotation and flexion-extension was seen with increased decompression but not to a significant level [13]. Gao et al. found that there was no significant change in the intersegmental angles with distraction of 3 mm [15]. With limited effect on spinal movements, pedicle distraction has potential to be a stable method of decompression. More study is required to evaluate altered biomechanics, sagittal balance in single and multi-level distraction to fully understand the impact of the technique.

The authors recognise that performing the technique ex vivo and in cadavers is not equivalent to the operation room. Studies have shown the improved accuracy of pedicle screw placement with navigation and guidance techniques [16,17]. Performing the osteotomy from within the drilled pedicle channel has been shown to be possible. Using C-arm guidance accurate osteotomy could be performed at 2 mm from the posterior vertebral body to within 0.3 mm of accuracy [18]. While not replicating the full operative environment, the above studies indicate the efficacy of both pedicle screw and osteotomy techniques with a minimally invasive approach.

We are the first to our knowledge to document the anatomy and soft tissues involved in an osteotomy of the pedicle and its effects on surrounding structures. We noted the safety of the dura and exiting nerve root when performing an osteotomy at the midpoint of the pedicle. The most pressing relation is that of the exiting nerve root. The dura was tethered to the posterior arch, yet up to 6 mm of distraction caused no damage or tearing to our specimen. The major limiting factors to distraction arise from the facet joints and posterior ligamentous structures. While we distracted up to 6 mm, some decompression was noted at lower levels of distraction. Dependent on the pathology and degree of decompression needed, smaller amounts of distraction may be appropriate imparting less stress on the posterior segment.

## Conclusion

For each incremental increase in pedicle distraction the area of both the spinal and intervertebral foramen increases. Distraction of the pedicles is a potential technique for alleviating spinal stenosis and nerve root impingement through decompression and enlargement of the rigid bony canals.

From our cadaveric study pedicle distraction appears to cause little damage to the dura and surrounding soft tissues. Further work needs to be done on the biomechanics and potential effects of the distraction force.

## Conflict of interest

The authors declare that they have no conflicts of interest in relation to this article.
